# miRNA Expression Profiling Uncovers a Role of miR-139-5p in Regulating the Calcification of Human Aortic Valve Interstitial Cells

**DOI:** 10.3389/fgene.2021.722564

**Published:** 2021-10-22

**Authors:** Fan Zhang, Naixuan Cheng, Yingchun Han, Congcong Zhang, Haibo Zhang

**Affiliations:** ^1^ Department of Cardiac Surgery, Beijing Anzhen Hospital, Capital Medical University, Beijing, China; ^2^ Key Laboratory of Remodeling-Related Cardiovascular Diseases, Ministry of Education, Beijing Institute of Heart, Lung and Vascular Diseases, Beijing, China

**Keywords:** calcific aortic valve disease, miR-139-5p, aortic valve interstitial cells, osteogenesis, Wnt/b-catenin

## Abstract

Calcific aortic valve disease (CAVD) is the most common structural heart disease, and the morbidity is increased with elderly population. Several microRNAs (miRNAs) have been identified to play crucial roles in CAVD, and numerous miRNAs are still waiting to be explored. In this study, the miRNA expression signature in CAVD was analyzed unbiasedly by miRNA-sequencing, and we found that, compared with the normal control valves, 152 miRNAs were upregulated and 186 miRNAs were downregulated in calcified aortic valves. The functions of these differentially expressed miRNAs were associated with cell differentiation, apoptosis, adhesion and immune response processes. Among downregulated miRNAs, the expression level of miR-139-5p was negatively correlated with the osteogenic gene *RUNX2,* and miR-139-5p was also downregulated during the osteogenic differentiation of primary human aortic valve interstitial cells (VICs). Subsequent functional studies revealed that miR-139-5p overexpression inhibited the osteogenic differentiation of VICs by negatively modulating the expression of pro-osteogenic gene *FZD4* and *CTNNB1*. In conclusion, these results suggest that miR-139-5p plays an important role in osteogenic differentiation of VICs *via* the Wnt/β-Catenin pathway, which may further provide a new therapeutic target for CAVD.

## Introduction

Calcific aortic valve disease (CAVD), which results in aortic valve stenosis, affects 25% of the population >65 years of age (Coffey et al., 2014; Lindman et al., 2016). In developing countries, CAVD represents a major cause for surgical valve replacement. CAVD confers a high clinical and economic burden, because no effective pharmacological therapy exists (Hutcheson et al., 2014). The disease progression is rapid and, although initially indolent, it results in heart failure and premature death if left untreated. Owing to its high morbidity and mortality, a strong incentive exists to identify the key molecular drivers contributing to the development of this disease, which could provide a new target for clinical treatment in its earlier stages before cardiac damage.

CAVD is marked by inflammatory infiltration, fibrotic extracellular matrix (ECM) synthesis by activated valve interstitial cells (VICs), increased leaflet thickening and stiffness and calcific mineral deposition (Peeters et al., 2018). The resultant pathological remodeling ultimately impairs valve movement and obstructs blood flow across the narrowing valve orifice. In healthy valves, VICs comprise mostly quiescent fibroblasts that maintain valvular homeostasis and physiological leaflet mechanical properties. Under pathological stimuli, VICs undergo differentiation toward osteoblastic phenotypes as a result of newly acquired expression of cytoskeletal or osteogenic genes (*RUNX2*, *ALPP*, *BMP2*, *BGLAP*, etc.) (Rutkovskiy et al., 2017). However, the pathogenic mechanisms that trigger their maladaptive differentiation *in vivo* are unclear.

MicroRNA (miRNA) is a type of small RNA that is generally 22–25 bases in length (Lagos-Quintana et al., 2001). miRNA binds to the 3′-untranslated regions (UTR) of the target mRNA in the cytoplasm by sequence complementarity, which inhibits the translation of the target mRNA or causes the degradation of the target mRNA (Miranda et al., 2006; Sun and Lai, 2013). Compared with normal human aortic valves, the miRNA expression profiles in calcified aortic valve tissues have changed (Wang et al., 2017). Several miRNAs [miR-204-5p (Yu et al., 2018; Song et al., 2019), miR-143 (Fiedler et al., 2019), miR-125b (Ohukainen et al., 2015), miR-34a (Toshima et al., 2020), etc.] have been reported to participate in the osteogenic differentiation of VICs and valve calcification.

In this study, to identify the key miRNAs during aortic valve calcification, we explored the miRNA expression signature in CAVD by miRNA-sequencing and combined analyzed with a published transcriptome data (https://cics.bwh.harvard.edu/multiomics_databases) (Schlotter et al., 2018). Among the differentially expressed miRNAs (DEMs), miR-139-5p was downregulated miRNA in calcified aortic valves and primary human aortic VICs. Subsequent functional studies revealed that miR-139-5p overexpression results in inhibition of VICs osteogenic differentiation by negatively modulating the expression of pro-osteogenic gene *FZD4* and *CTNNB1*. Therefore, our study provided new insights into the function of miR-139-5p in the pathogenesis of CAVD.

## Materials and Methods

### Clinical Samples

A total of nine calcified aortic valves (CAVs) (exclusion criteria: rheumatic aortic valvulopathy, infective endocarditis, congenital valve disease, bicuspid aortic valve) from patients with CAVD and 8 normal aortic valves (CONs) from patients with heart transplantation were obtained at the Department of Cardiovascular Surgery, Anzhen Hospital, affiliated to Capital Medical University. The clinical characteristics of all samples are listed in Table 1. All the studies involving human samples complied with the Declaration of Helsinki and were approved by the Ethics Committee of Anzhen Hospital, affiliated to Capital Medical University. Written informed consent was obtained from the patients before surgery.

**TABLE 1:** Clinical characteristics in patients with normal valves and CAV.

	Normal control (*n* = 8)	CAV (*n* = 9)	*p*-value
Male, *n* (%)	7 (87.5)	6 (66.7)	0.577
Age, years	50.3 ± 7.9	58.4 ± 11.6	0.104
BMI, kg/m^2^	25.8 ± 3.9	27.9 ± 3.7	0.235
Coronary artery disease, %	2 (25)	2 (22.2)	1.000
Dyslipidemia/hypercholesterolemia, %	3 (37.5)	2 (22.2)	0.620
Hypertension, %	5 (62.5)	7 (77.8)	0.620
Smoking history, *n* (%)	2 (25)	5 (55.6)	0.3348
Aortic valve area, cm^2^	NA	0.77 ± 0.16	—
Mean gradient, mmHg	NA	41.2 ± 15.6	—
Aortic valve peak flow velocity (m/s)	NA	4.7 ± 0.6	—

Continuous variables are expressed as means ± SD. Dichotomous variables are expressed as percentage.

### RNA Extraction and miRNA-Sequencing

Total RNAs were extracted from aortic valves and cells using TRIzol Reagent (Life technologies) as described previously (Han et al., 2021). RNA from five calcified aortic valves and 3 normal aortic valves were used for miRNA-sequencing. After using Agilent 2100 Bio analyzer (Agilent RNA 6000 Nano Kit) to do the total RNA sample quality control, cDNA library was constructed and sequenced on DNBseq platform. DEMs were identified by comparing the gene-level fragments kilobase of exon model per million mapped reads (FKPM) between two groups and selected by using the criteria with at least a twofold change of FPKM and false discovery rate (FDR) value <0.001. The target genes of DEMs were predicted with RNAhybrid, miRanda and TargetScan database and further function analysis by gene ontology (GO) biological process (BP) term and Kyoto Encyclopedia of Genes and Genomes (KEGG) pathway enrichment analysis was performed using DAVID Bioinformatics Resources 6.8 (https://david.ncifcrf.gov/). All the raw data can be accessed in the GEO database (GSE171208).

### Real-Time PCR

For miRNAs, the isolated total RNAs were reverse transcribed into complementary cDNAs and qRT-PCR analysis using Bulge-Loop miRNA qRT-PCR kit (Ribobio, C10211-2 Guangzhou, China) and the Bulge-Loop primer of hsa-miR-374c-3p (Ribobio, miRA1000644), hsa-miR-181a-2-3p (Ribobio, miRA1000331), hsa-miR-490-5p (Ribobio, miRA1000817), hsa-miR-204-5p (Ribobio, miRA0000265), hsa-miR-1180-3p (Ribobio, miRA1000016), hsa-miR-149-5p (Ribobio, miRA1000346), hsa-miR-139-5p (Ribobio, miRA1000053), and U6 (Ribobio, miRAN0002-1-100). U6 was used as an internal control.

For mRNAs, the isolated total RNAs were reversely transcribed into complementary cDNAs using GoScript™ Reverse Transcriptase Kit (Promega, A5001) and then real-time PCR analysis was performed using SYBR Green PCR Master Mix Reagent Kit (TaKaRa), with *GAPDH* used as an internal control. Data were presented as values calculated by the 2^ΔΔt^ method. The sequences of primers are as follows:

*ALPP*: 5′-GTG​AAC​CGC​AAC​TGG​TAC​TC-3′, 5′-GAG​CTG​CGT​AGC​GAT​GTC​C-3′; *RUNX2*: 5′-TGG​TTA​CTG​TCA​TGG​CGG​GTA-3′, 5′-TCT​CAG​ATC​GTT​GAA​CCT​TGC​TA-3′; *BMP3*: 5′-TGA​CAT​CGC​TAA​CCA​AGT​CTG​A-3′, 5′-TGA​GGG​TCC​ATG​CAG​AAA​GAT-3′; *BGLAP*: 5′-CAC​TCC​TCG​CCC​TAT​TGG​C-3′, 5′-CCC​TCC​TGC​TTG​GAC​ACA​AAG-3′; *CTNNB1*: 5′-AGC​TTC​CAG​ACA​CGC​TAT​CAT-3′, 5′-CGG​TAC​AAC​GAG​CTG​TTT​CTA​C-3’; *FZD4*: 5′-GTG​TCA​CTC​TGT​GGG​AAC​CAA-3′, 5′-GGC​TGT​ATA​AGC​CAG​CAT​CAT-3’; *GAPDH*: 5′-GGA​GCG​AGA​TCC​CTC​CAA​AAT-3′, 5′-GGC​TGT​TGT​CAT​ACT​TCT​CAT​GG-3′.

### VECs and VICs Isolation, Culture, and Osteogenic Differentiation

Primary valve endothelial cells (VECs) and VICs were isolated from normal aortic valve leaflets according to a previously reported protocol (Li et al., 2017a). After enzymatic digestion of the aortic valve leaflets with 1 mg/ml collagenase (Type I) at 37°C for 30 min, the endothelial cells were removed from valve leaflets by cell scraper and collected in 1 × PBS buffer. After centrifugation for 5 min, VECs were resuspended in ECM with 10% heat-inactivated FBS and 1% penicillin/streptomycin and cultured in 37°C humidified atmospheres at 5% CO_2_. Then, the remaining left leaflets were minced and further digested with collagenase (Type I) at 37°C for 1–2 h. After enzymatic digestion, the cell suspension was filtered with a 70 μm cell strainer and VICs were cultured in standard DMEM with 10% heat-inactivated FBS and 1% penicillin/streptomycin in a 37°C humidified atmosphere at 5% CO_2_.

VECs and VICs were identified by immunofluorescence staining. After fixation with 4% paraformaldehyde and blocking with 5% bovine serum albumin, primary antibody [rabbit polyclonal antibody to CD31 (Abcam, ab28364, 1:100 diluted) or rabbit polyclonal antibody to Periostin (Santa Cruz, sc-67233, 1:50 diluted)] was incubated at 4°C overnight and then a second antibody [Alexa Fluor 555 donkey anti-rabbit (Life Technologies, A31572, 1:500 diluted) or Alexa Fluor 488 donkey anti-rabbit (Life Technologies, A21206, 1:500 diluted)] was incubated for 1 h at room temperature. The nuclei were stained with 4′,6-diamidino-2-phenylindole (DAPI) (Abcam).

Calcification of VICs was induced by osteogenic medium [OM, 50 μM ascorbic acid phosphate (Sigma), 100 nM dexamethasone (Sigma) and 10 μM β-glycerophosphate (Sigma)]. VICs were cultured in OM for 14 days with a replacement every 2–3 days. Alizarin red staining was used to access the matrix calcium deposition after fixation with 10% formalin.

### Transfection of VICs With miRNA Mimics

VICs were transfected at a confluency of 70–80% in a 12-well plate after seeding. micrON^TM^ hsa-miR-139-5p mimic (50 nM) or negative controls (micrON^TM^ miRNA mimic NC #22, Ribobio) were transfected using Lipofectamine 3000 according to the manufacturer’s protocol (Invitrogen). The transfection efficiency was examined by real-time PCR after 48 h of transfection. During the osteogenic differentiation induction of VICs, miRNA mimics were added at the same time of OM replacement.

### Statistical Analysis

Data processing was performed using SPSS21.0 software. The measurement data were expressed as mean ± standard deviation and the unpaired *t*-test was used for comparison between two groups and one-way ANOVA was used for comparison in three groups. Spearman’s correlation test was used for correlation analysis. *p* < 0.05 was considered to have a statistically significant different between groups.

## Results

### miRNA Expression Profile was Altered During Aortic Valve Calcification

**FIGURE 1:**
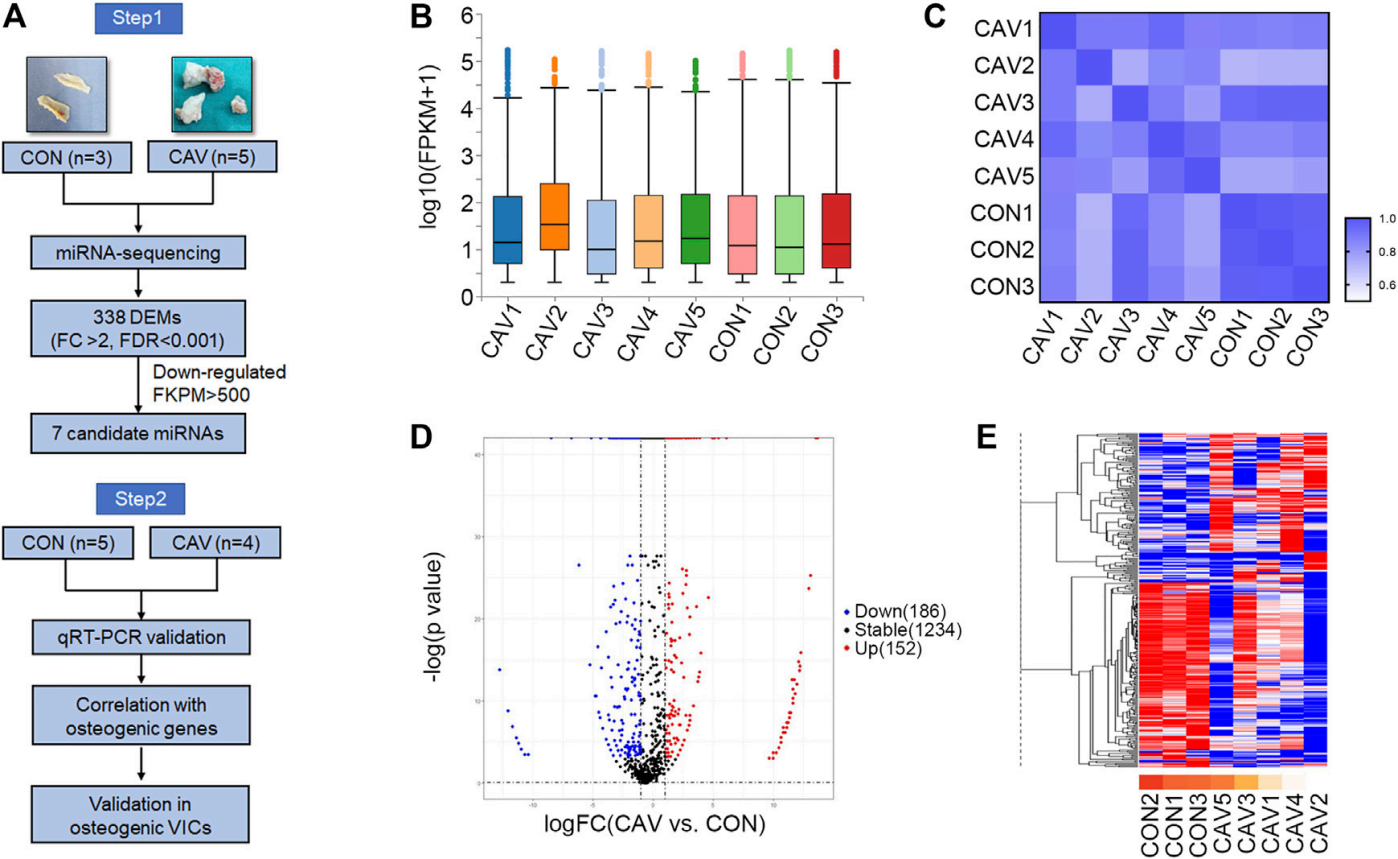
miRNA expression profile was altered during aortic valve calcification. **(A)** Schematic description of the workflow illustrating the two-stage-approach involving independent samples for discovery and validation. **(B)** Overall count distribution of miRNAs in each sample in the RNA-sequencing experiment. **(C)** Pearson correlation coefficient of overall miRNA expression levels between every two samples. **(D)** Volcano plot revealing miRNA-sequencing results comparing CON (*n* = 3 biologically independent samples) versus CAV (n = 5 biologically independent samples). Individual miRNAs are displayed by the *p*-value and the corresponding fold change. **(E)** Heatmap of the 338 differentially expressed miRNAs. Red color indicated higher expression level; blue color indicated lower expression level. CON, control; CAV, calcified aortic valves.

To explore the DEMs, total RNAs from three control aortic valves and five calcified aortic valves were analyzed by genome-wide miRNA expression profiling using DNBseq sequencing platform (Figure 1A). The average alignment ratio of the sample comparison genome (Homo_sapiens_9606.NCBI.GCF_000001405.38_GRCh38.p12. v1904) was 93.48%. A total of 1,572 miRNAs were detected. The boxplots in Figure 1B showed comparable read count distribution across all eight samples. Based on the analysis of Pearson correlation coefficient of overall miRNA expression levels between every two samples, the eight samples could be divided into two groups, which is consistent with the original groups (Figure 1C). Then, significant DEMs were identified as those with a change fold >2.0 and FDR <0.001. Compared with the control group, 152 miRNAs were upregulated and 186 miRNAs were downregulated in calcified aortic valves (Figure 1D, Supplementary Excel S1). The expression patterns of the DEMs were shown in the hierarchical clustering heatmap (Figure 1E).

### Pathway Analysis of the Predict Target Genes of Differentially Expressed miRNAs

**FIGURE 2:**
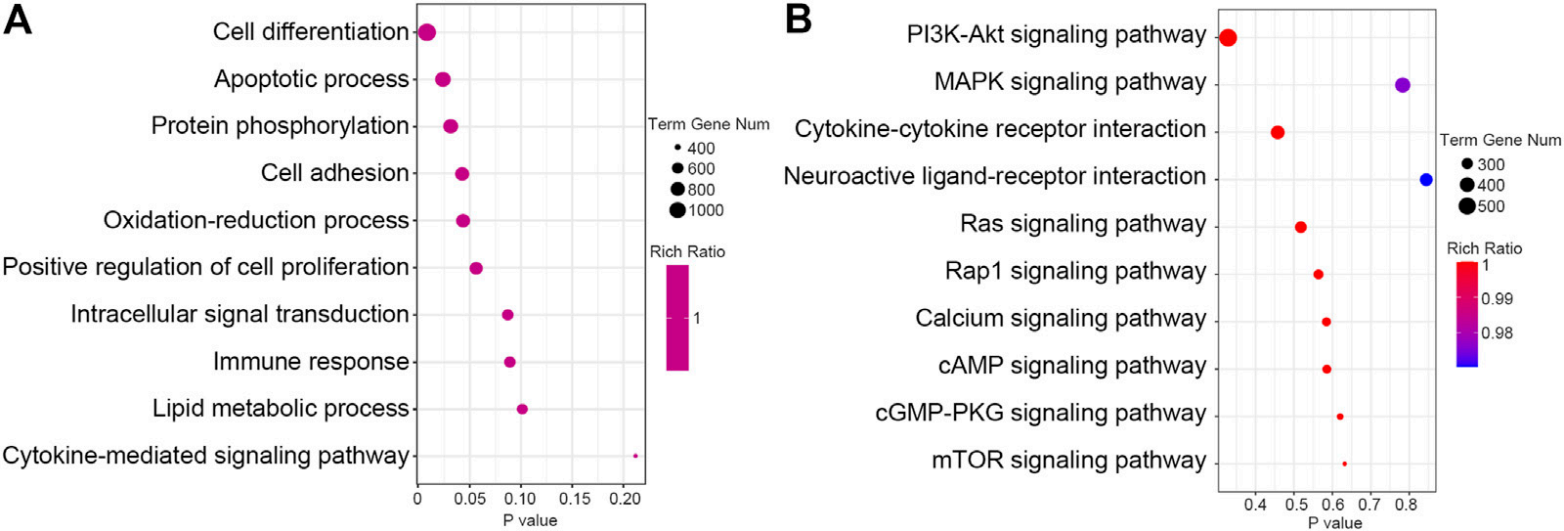
Pathway analysis of the predict target genes of differential expressed miRNAs. **(A)** The GO biological process (BP) function of the predict target genes of differential expressed miRNAs was analyzed and the top 10 terms were listed. **(B)** KEGG pathway of the predict target genes of differential expressed miRNAs was analyzed and the top 10 pathways were listed.

Identification the function enrichment of predicted targets may help to understand the biological role of these DEMs. We used RNAhybrid, miRanda and TargetScan database to predict the target mRNAs for these DEMs. Through GO BP enrichment analysis, we found that the target genes of DEMs were associated with cell differentiation, apoptosis process, cell adhesion, oxidation–reduction process and immune response (Figure 2A). Through the KEGG pathway enrichment analysis, we found that the target genes of DEMs were significantly enriched in a few signaling pathways, especially the phosphatidylinositol 3ʹ-kinase (PI3K)–protein kinase B (AKT) signaling pathway, mitogen-activated protein kinase (MAPK) signaling pathway, and cytokine–cytokine receptor interaction pathway (Figure 2B).

### Dysregulated Signaling Pathway in Calcified Aortic Valves

Then, we further analyzed the altered signaling pathway in a previous published data (https://cics.bwh.harvard.edu/multiomics_databases) (Schlotter et al., 2018), which compared the transcriptome of three non-calcified aortic valves and three calcified aortic valves. Differentially expressed genes (DEGs) were identified as those with a change fold >2.0. Compared with the non-calcified group, 1,235 genes were upregulated and 544 genes were downregulated in calcified aortic valves and the hierarchical clustering heatmap is shown in Supplementary Figure S1A. Through GO BP enrichment analysis, we found that the upregulated genes were associated with immune response, chemokine-mediated signaling pathway, T-cell activation, positive regulation of extracellular signal-regulated kinase (ERK)1/2 cascade and extracellular matrix disassembly (Supplementary Figure S1B). The downregulated genes were associated with negative regulation of canonical Wnt signaling pathway, muscle contraction, negative regulation of cell proliferation and ossification (Supplementary Figure S1C). Through the KEGG pathway enrichment analysis, the upregulated DEGs were significantly enriched in cytokine–cytokine receptor interaction, osteoclast differentiation, cell adhesion molecules and PI3K–AKT signaling pathway (Supplementary Figure S1D). The downregulated DEGs were significantly enriched in protein digestion and absorption, Wnt signaling pathway, TGF-beta signaling pathway and Hippo signaling pathway (Supplementary Figure S1E). These results have shown that the function enrichment of DEMs in our miRNA-seq had some similarities with the function enrichment of DEGs in RNA-seq.

### The Verification of Downregulated Candidate miRNAs in Calcified Aortic Valves

**FIGURE 3:**
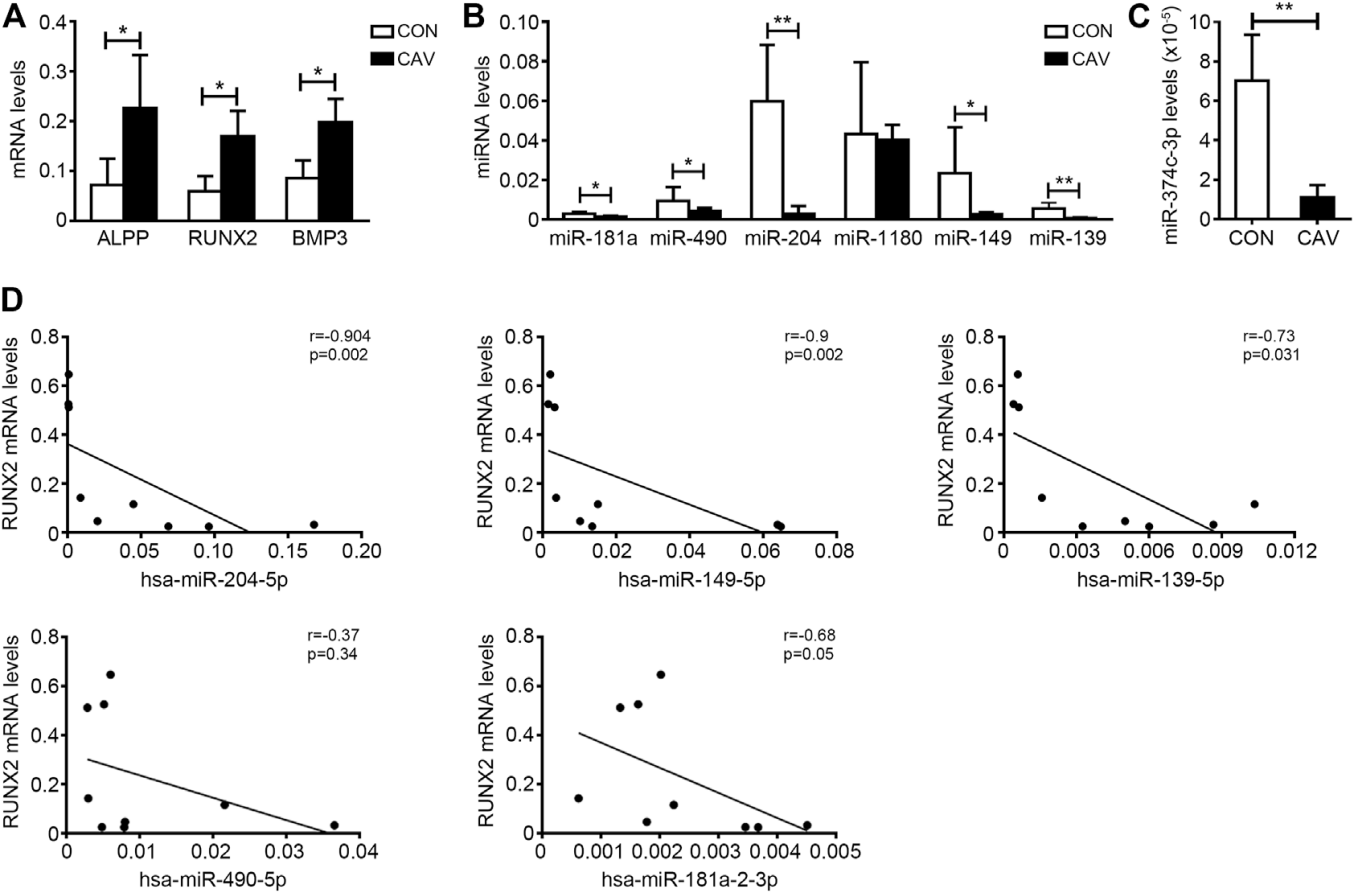
The verification of downregulated candidate miRNAs in calcified aortic valves. **(A)** The expression levels of osteogenic genes *ALPP*, *RUNX2* and *BMP3* in valves from CON patients (*n* = 5 biologically independent samples) and CAV patients (*n* = 4 biologically independent samples) were accessed by qRT-PCR and normalized with *GAPDH*. **(B,C)** The expression levels of hsa-miR-181a-2-3p (miR-181a), hsa-miR-490-5p (miR-490), hsa-miR-204-5p (miR-204), hsa-miR-1180-3p (miR-1180), hsa-miR-149-5p (miR-149), hsa-miR-139-5p (miR-139) **(B)** and hsa-miR-374c-3p **(C)** in valves from CON patients (*n* = 5 biologically independent samples) and CAV patients (*n* = 4 biologically independent samples) were accessed by qRT-PCR and normalized with U6. **(D)** Spearman’s correlation test was used to analyze the correlation of the expression of hsa-miR-204-5p, hsa-miR-149-5p, hsa-miR-139-5p, hsa-miR-181a-2-3p and hsa-miR-490-5p with the expression levels of *RUNX2* in aortic valves. Unpaired Student’s *t*-test, *p < 0.05, **p < 0.01. CON, control; CAV, calcified aortic valves.

We selected candidate miRNAs from the downregulated miRNAs for validation by real-time PCR. Among the top 30 miRNAs (ranked by change fold), there were 7 miRNAs (hsa-miR-374c-3p, hsa-miR-181a-2-3p, hsa-miR-490-5p, hsa-miR-204-5p, hsa-miR-1180-3p, hsa-miR-149-5p and hsa-miR-139-5p) highly expressed in normal valves (FKPM > 500) (Table 2). Then, we validated the expression of these miRNAs in another five normal valves and four calcific valves. The expression levels of osteogenic genes *ALPP*, *RUNX2*, *BMP3*, *BGLAP*, *FZD4* and *CTNNB1* were higher in calcific valves than normal valves (Figure 3A and Supplementary Figure S2). Compared to the normal valves, six miRNAs (hsa-miR-374c-3p, hsa-miR-181a-2-3p, hsa-miR-490-5p, hsa-miR-204-5p, hsa-miR-149-5p, and hsa-miR-139-5p) were significantly downregulated in calcified aortic valves, while the expression levels of hsa-miR-1180-3p were not different between two groups (Figures 3B,C). Because the expression level of hsa-miR-374c-3p in valves was lower than the other five miRNAs, we removed it from the candidate miRNAs. Among the five candidate miRNAs, it was previously reported that the expression of miR-204-5p was decreased in calcified aortic valves and inhibited the osteogenic differentiation of VICs and aortic valve calcification (Yu et al., 2018; Song et al., 2019). Then, we calculated the correlation of five candidate miRNAs with the osteogenic genes *RUNX2*. miR-204-5p, miR-149-5p, and miR-139-5p exhibited significant negative correlations with osteogenic genes *RUNX2* (Figure 3D). These data indicated that, besides miR-204-5p, miR-149-5p and miR-139-5p may also be the key miRNAs in the development of CAVD.

**TABLE 2:** Top 30 downregulated miRNA in valves from CAVD.

Gene ID	Mean CON expression	Mean CAV expression	log_2_ change fold (CAV/CON)	Q value (CON vs. CAV)
hsa-miR-3184-3p	498.333	1.4	−8.47554	0
**hsa-miR-374c-3p**	**12,591**	**112.2**	**−6.81018**	**0**
hsa-miR-1224-5p	14.333	0.2	−6.1632	1.05E-11
hsa-miR-3622b-3p	70.667	2	−5.14296	6.47E-52
hsa-miR-488-3p	62.667	3	−4.38467	3.36E-41
**hsa-miR-181a-2-3p**	**607.667**	**29.8**	**−4.3499**	**0**
hsa-miR-208a-3p	12	0.8	−3.90689	3.34E-08
hsa-miR-490-3p	10.667	0.8	−3.73701	6.92E-07
hsa-miR-202-5p	103.333	8.2	−3.65553	1.02E-56
hsa-miR-429	248.333	20.4	−3.60563	1.47E-132
hsa-miR-1298-5p	16.333	1.4	−3.54429	1.74E-09
hsa-miR-509-3p	69	6.2	−3.47626	8.01E-36
**hsa-miR-490-5p**	**2611.667**	**241.4**	**−3.43547**	**0**
hsa-miR-1468-5p	277	27.4	−3.33764	8.17E-136
hsa-miR-4796-5p	18	1.8	−3.32193	1.25E-09
hsa-miR-4775	19.333	2	−3.27299	1.40E-10
hsa-miR-3622a-5p	30.667	3.2	−3.26054	4.75E-16
hsa-miR-219b-5p	18	2	−3.16993	7.69E-10
hsa-miR-1299	99.667	11.4	−3.12808	4.79E-45
hsa-miR-134-3p	17.333	2	−3.11545	1.25E-08
**hsa-miR-204-5p**	**19,798.33**	**2309.6**	**−3.09966**	**0**
**hsa-miR-1180-3p**	**3436.667**	**414.4**	**−3.05191**	**0**
hsa-miR-4662a-5p	173	21	−3.04231	2.17E-75
**hsa-miR-149-5p**	**1550.333**	**190.2**	**−3.02699**	**0**
**hsa-miR-139-5p**	**4220.667**	**528.2**	**−2.99831**	**0**
hsa-miR-3065-3p	13.667	1.8	−2.92463	1.71E-06
hsa-miR-935	28.667	3.8	−2.91532	3.98E-13
hsa-miR-200b-3p	20.667	3	−2.78429	1.08E-08
hsa-miR-1247-5p	419.667	61.8	−2.76357	2.46E-156
hsa-miR-548ah5p	14	2.2	−2.66985	7.01E-06

Ranked by log_2_ change fold; Bold: FKPM > 500 in normal valves (CON).

### Both miR-149-5p and miR-139-5p Were Downregulated in Osteogenically Differentiated VICs

**FIGURE 4:**
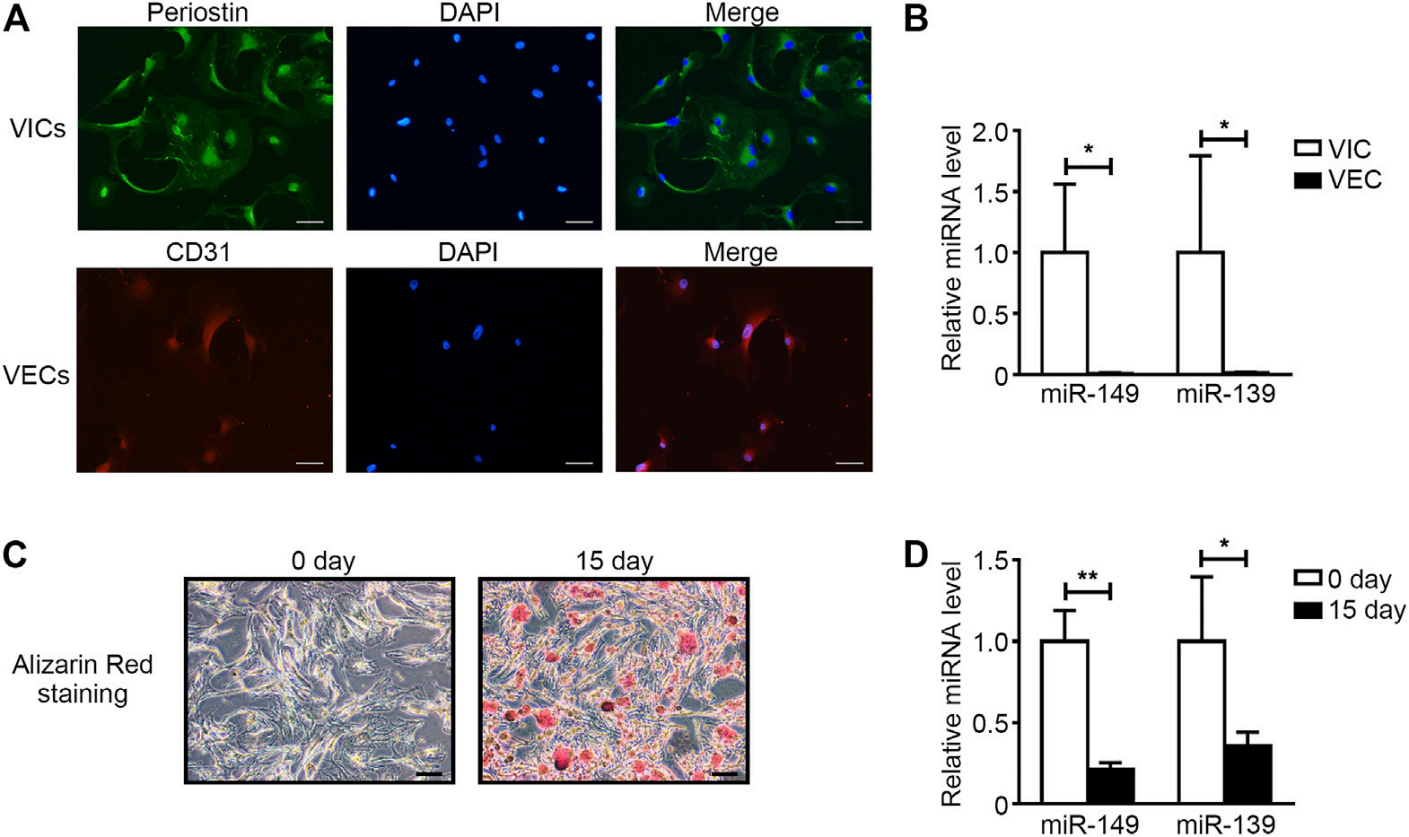
Both miR-149-5p and miR-139-5p were downregulated in osteogenic differentiated VICs significantly. **(A)** Primary cultured VICs and VECs were immunofluorescence stained with Periostin (green) and CD31 (red), respectively and the nucleus was stained with DAPI (blue) (the scale bar = 50 μm). **(B)** The expression levels of hsa-miR-149-5p (miR-149) and hsa-miR-139-5p (miR-139) in VECs and VICs were accessed by qRT-PCR and normalized with U6 (*n* = 4 samples from independent wells in each group). **(C)** Alizarin red staining was used to access the matrix calcium deposition in VICs at 15 days after OM induction. **(D)** The expression levels of hsa-miR-149-5p and hsa-miR-139-5p in control and osteogenic differentiated VICs were accessed by qRT-PCR and normalized with U6 (n = 4 samples from independent wells in each group). Unpaired Student’s *t*-test, *p < 0.05, **p < 0.01.

To confirm the cell location of miR-149-5p and miR-139-5p, we cultured the primary VICs and VECs and compared the expression levels of miR-149-5p and miR-139-5p in VICs and VECs (Figure 4A and Supplementary Figure S3). Both the expression levels of miR-149-5p and miR-139-5p were higher in VICs than that in VECs (Figure 4B). Then, we induced the osteogenic differentiation of VICs by condition medium (Figure 4C) and compared the expression levels of miR-149-5p and miR-139-5p in primary VICs and osteogenically differentiated VICs. We found that miR-149-5p and miR-139-5p decreased 4.66-fold and 2.79-fold, respectively, in osteogenically differentiated VICs (Figure 4D).

### miR-139-5p Overexpression Could Inhibit the Osteogenic Differentiation of VICs

**FIGURE 5:**
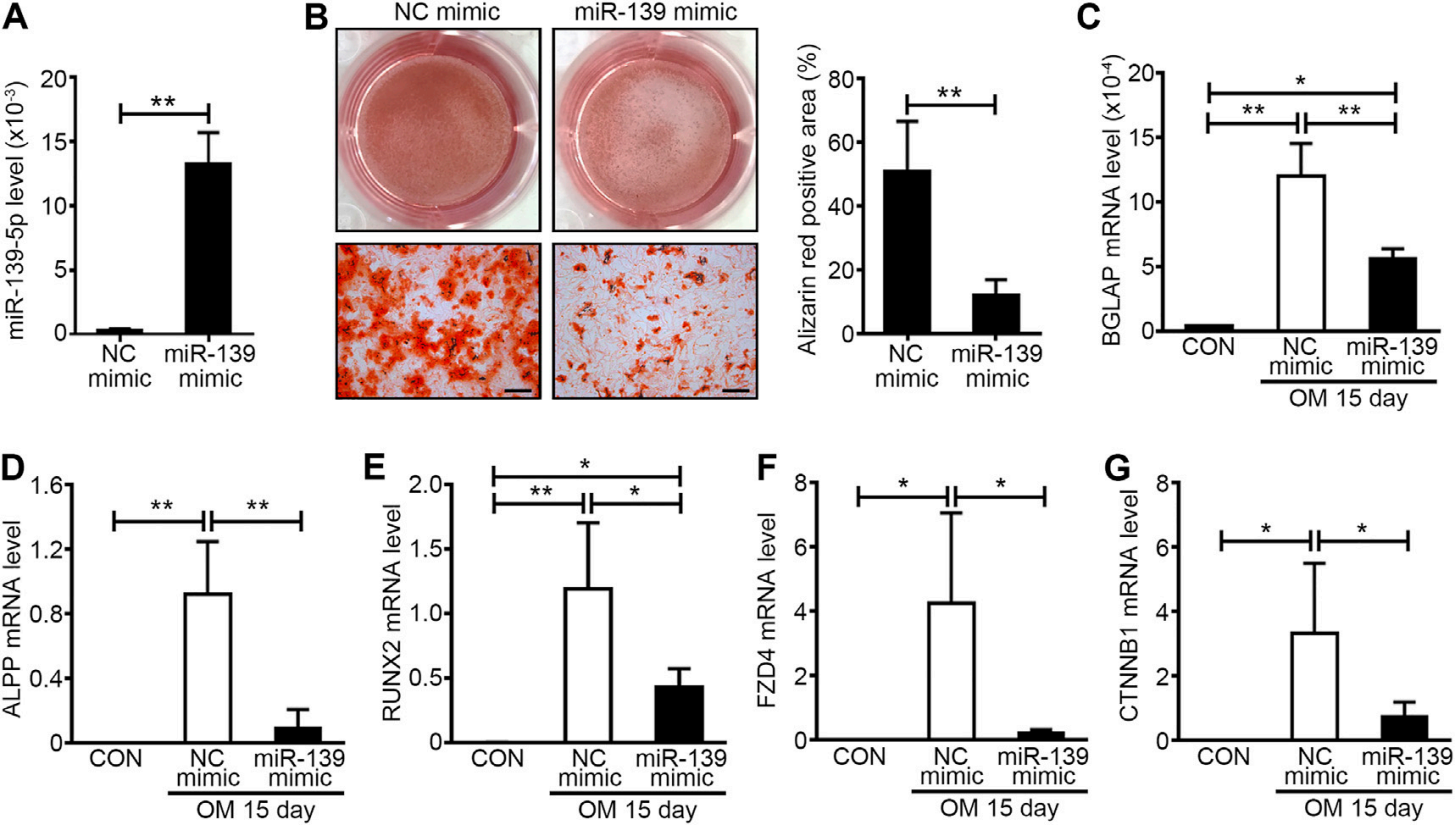
miR-139-5p overexpression could inhibit the osteogenic differentiation of VICs. **(A)** The expression levels of miR-139-5p in negative control (NC) mimics and miR-139-5p mimics (miR-139 mimic) transfected VICs were accessed by qRT-PCR and normalized with U6 (*n* = 4 samples from independent wells in each group). Unpaired Student’s *t*-test, **p < 0.01. **(B)** Alizarin red staining was used to access the matrix calcium deposition in NC mimics and miR-139-5p mimics transfected VICs at 15 days after OM induction and the right graph was the percentage of Alizarin red positive area per field (n = 3 wells in each group and three to four fields per well were counted, the scale bar = 200 μm). Unpaired Student’s *t*-test, **p < 0.01. **(C–E)** The expression levels of *BGLAP* **(C)**, *ALPP* **(D)** and *RUNX2* **(E)** in NC mimics and miR-139-5p mimics transfected VICs were accessed by qRT-PCR and normalized with *GAPDH* (*n* = 4 samples from independent wells in each group). **(F,G)** The expression levels of *FZD4* **(F)** and *CTNNB1* **(G)** in NC mimics and miR-139-5p mimics transfected VICs were accessed by qRT-PCR and normalized with *GAPDH* (*n* = 4 samples from independent wells in each group). One-way ANOVA, *p < 0.05, **p < 0.01.

It was reported that miR-139-5p could repress the osteogenesis of mesenchymal stem cells *via* targeting Wnt/β-Catenin signaling pathway (Long et al., 2017). For the importance of Wnt/β-Catenin signaling pathway to osteogenesis of VICs and aortic valve calcification (Khan et al., 2020; Albanese et al., 2017), we focused on the effects of miR-139-5p on osteogenesis the VICs in further study. miR-139-5p mimics were transfected to VICs during osteogenic differentiation, the NC mimics as negative control. The expression of miR-139-5p in VICs transfected with miR-139-5p mimics was higher than that in NC mimics transfected VICs at 15 days after osteogenic induction, indicating that the miR-139-5p mimics were successfully transfected into VICs (Figure 5A). Alizarin Red staining showed that calcium deposition in VICs with the miR-139-5p mimics was less than that in VICs with NC mimics (Figure 5B). At the same time, the expression levels of osteogenic genes *BGLAP*, *ALPP* and *RUNX2* in VICs were also decreased by miR-139-5p overexpression (Figures 5C–E). *FZD4* and *CTNNB1*, the key genes of Wnt/β-Catenin pathway, were the reported target genes of miR-139-5p (Long et al., 2017). We found that the expression levels of *FZD4* and *CTNNB1* were increased in osteogenically differentiated VICs, while they were repressed by miR-139-5p overexpression (Figures 5F,G). Therefore, these data demonstrated that miR-139-5p could inhibit the osteogenic differentiation of VICs by targeting the Wnt/β-Catenin pathway.

## Discussion

miRNAs participate in the pathology of various cardiovascular diseases. By miRNA-seq, we found that the miRNA profile was altered in calcified aortic valves. Among the downregulated miRNAs, miR-139-5p could inhibit the osteogenic differentiation of VICs by target Wnt/β-Catenin pathway associated genes *FZD4* and *CTNNB1*.

Several studies have investigated the miRNA profile alternation in CAVD by quantitative RT-PCR based screening or microarray in the past decades. However, the detection throughput and sensitivity of those methods are relatively low and only hundreds of miRNAs can be detected. In one study, only 92 miRNAs were identified as DEMs with a change fold >2.0. Among the 92 DEMs, 53 miRNAs were downregulated and 39 were upregulated in aortic tissue from CAVD patients (Wang et al., 2017). In another study, 373 human miRNAs were examined by a quantitative RT-PCR based screening and found only 41 DEMs (Fiedler et al., 2019). In our study, miRNA-sequencing was used to the detect the DEMs in calcific aortic valves. Compared to microarray and RT-PCR-based screening, this method has the characteristics of high throughput and high sensitivity, which allows us to detect more miRNAs with higher accuracy. As a result, a total of 1,572 miRNAs were detected and there were 338 DEMs with a change fold >2.0 and FDR <0.001. Among the DEMs, 152 miRNAs were upregulated and 186 miRNAs were downregulated in calcified aortic valves, which indicated that more DEMs were found in this study. The significantly downregulated miRNAs found in the previous study, such as miR-125b (Ohukainen et al., 2015), miRNA-126 and Let-7 family (let-7a, let-7c, let-7d, let-7e and let-7f) (Wang et al., 2017), were also downregulated in our study. Other reported upregulated miRNAs, such as miR-34a (Toshima et al., 2020), miR-133a, miR-143 and miR-21 (Fiedler et al., 2019), were also upregulated in our study. Therefore, the method used in this study could contribute to fully understand miRNA profile alteration and the discovery of new functional targets in aortic valve calcification.

The pathology of calcific valve disease is an active and multifaceted condition involving lipoprotein deposition, inflammatory cell (including macrophages, T lymphocytes and mast cells) infiltration and cytokine secretion, osteogenic differentiation and apoptosis of VICs and ECM remodeling (Rutkovskiy et al., 2017; Peeters et al., 2018). In this study, by the functional and pathway enrichment analysis of the target genes of DEMs and DEGs, we found that both the DEMs and DEGs were associated with cell differentiation, apoptosis, adhesion and immune response and the associated pathways included the Wnt signaling pathway, ERK1/2 pathway, PI3K–AKT pathway, MAPK pathway and cytokine–cytokine receptor interaction pathway. Activation of the Wnt, PI3K–AKT and MAPK pathway promoted the osteogenic differentiation of VICs (Fang et al., 2014; Poggio et al., 2014; Xie et al., 2020). These results demonstrated that the DEMs in the miRNA-seq were indeed involved in the process of aortic valve calcification.

Since miRNA always acted as a negative regulator of transcription and there were more downregulated miRNAs than upregulated miRNAs and more upregulated mRNAs than downregulated mRNAs in calcified valves, we choose seven miRNAs from the downregulated miRNAs as candidates, which were abundant in normal valves. After validation in calcific valves and VICs and calculating the correlation with the osteogenic genes, we finally selected hsa-miR-139-5p from seven candidate miRNAs to verify its effect on VICs. The calcium deposition and the expression levels of *BGLAP*, *ALPP* and *RUNX2* were repressed by miR-139-5p overexpression, but these genes were not in the predicted target genes list. So, the effect of miR-139-5p on the osteogenic differentiation of VICs was mediated indirectly. The role of miR-139-5p in cell differentiation has been frequently reported. Mouse 3T3-L1 preadipocyte differentiation can be suppressed by miR-139-5p (Mi et al., 2015a). As a negative regulator in myogenesis, miR-139-5p has a great influence in myoblast differentiation by blocking the Wnt1-mediated Wnt/β-catenin signaling pathway (Mi et al., 2015b). In another study, miR-139-5p was able to repress the osteogenesis of mesenchymal stem cells *via* targeting Wnt/β-Catenin signaling pathway during the repair of bone (Long et al., 2017). *FZD4* (encodes Frizzled receptor isoforms 4) and *CTNNB1* (encodes β-Catenin), the reported target genes of miR-139-5p, are key molecular drivers in the Wnt/β-Catenin signaling pathway. Frizzled receptor 4, which is the receptor of Wnt family ligands, mediates the intracellular signaling activation with the lipoprotein-related peptide 5/6 (LRP5/6) co-receptors (Ahn et al., 2011). The typical Wnt/β-catenin signaling pathway activates the transcriptional activity of osteogenic genes (*RUNX2*, *ALPP*, etc.) by nuclear translocation of β-catenin (Calvisi et al., 2005; Park et al., 2011). LRP5 deficiency could prevent the calcification in the aortic valve under hypercholesterolemia condition (Rajamannan, 2011). In this study, we found that *FZD4* and *CTNNB1* were increased in osteogenic VICs and miR-139-5p could inhibit the expression the *FZD4* and *CTNNB1*. These data demonstrated that miR-139-5p could inhibit the osteogenic differentiation of VICs by targeting the Wnt/β-Catenin pathway.

In this study, we also found that miR-149-5p was downregulated in calcified valves and osteogenically differentiated VICs. In the previous study, interleukin-6 (IL-6) was reported as the target gene of miR-149-5p (Kong et al., 2018). The expression level of IL-6 was increased in calcified human aortic valves (Li et al., 2017b). Recombinant IL-6 can increase the expression of RUNX2 and osteopontin to promote the osteogenic differentiation of VICs; when IL-6 was inhibited by siRNA, the osteogenic differentiation of VICs was blocked *in vitro* (El Husseini et al., 2014; Grim et al., 2020). However, there is still no evidence for the function of miR-149-5p in osteogenic differentiation of VICs and CAVD and it could be explored in a future study.

In summary, our study fully described the changes in miRNA expression profile and its related pathological processes during aortic valve calcification by miRNA-seq. At the same time, we discovered that miR-139-5p plays an inhibiting role in the process of osteogenic differentiation, which may provide a new therapeutic target for aortic valve calcification.

## Data Availability

The original contributions presented in the study are publicly available. This data can be found here: https://www.ncbi.nlm.nih.gov/geo/, GSE171208.
