# Supplementary figures and images for: miRNA Expression Profiling Uncovers a Role of miR-139-5p in Regulating the Calcification of Human Aortic Valve Interstitial Cells
Source: Front Genet. 2021 Oct 22;12:722564. doi: 10.3389/fgene.2021.722564 (PMC8569802; doi:10.3389/fgene.2021.722564)

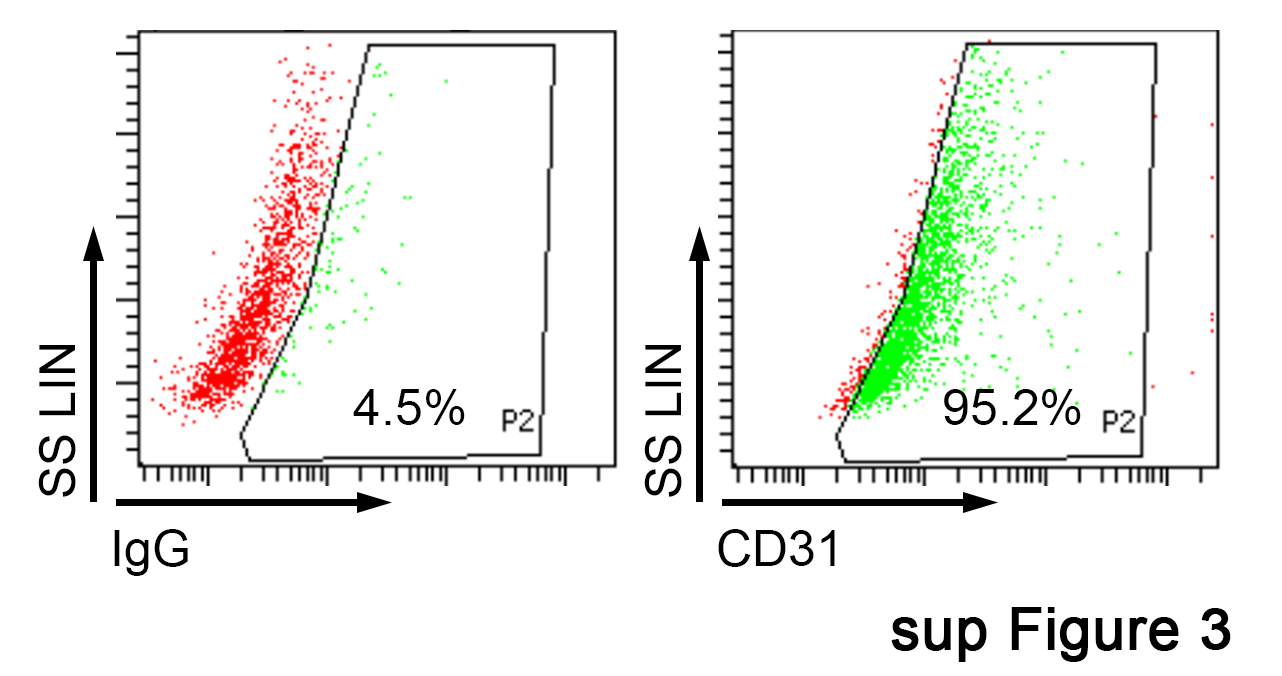

Supplement: Supplementary file 2 [file Image3.TIF]

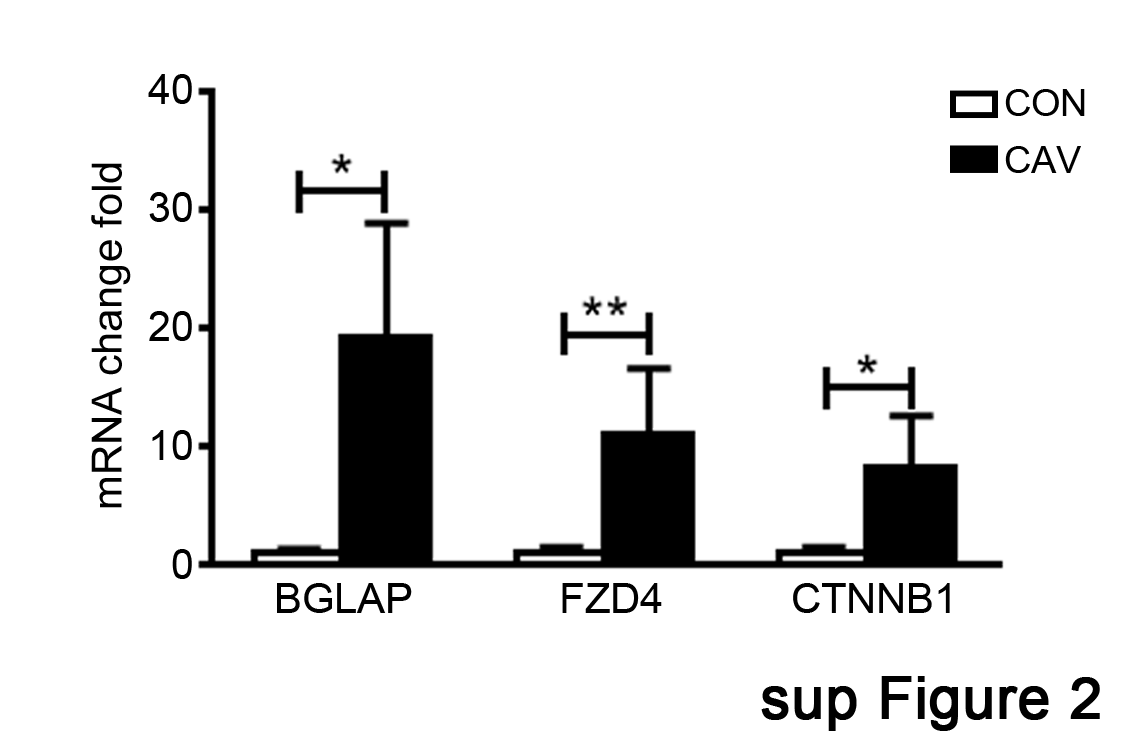

Supplement: Supplementary file 3 [file Image2.TIF]

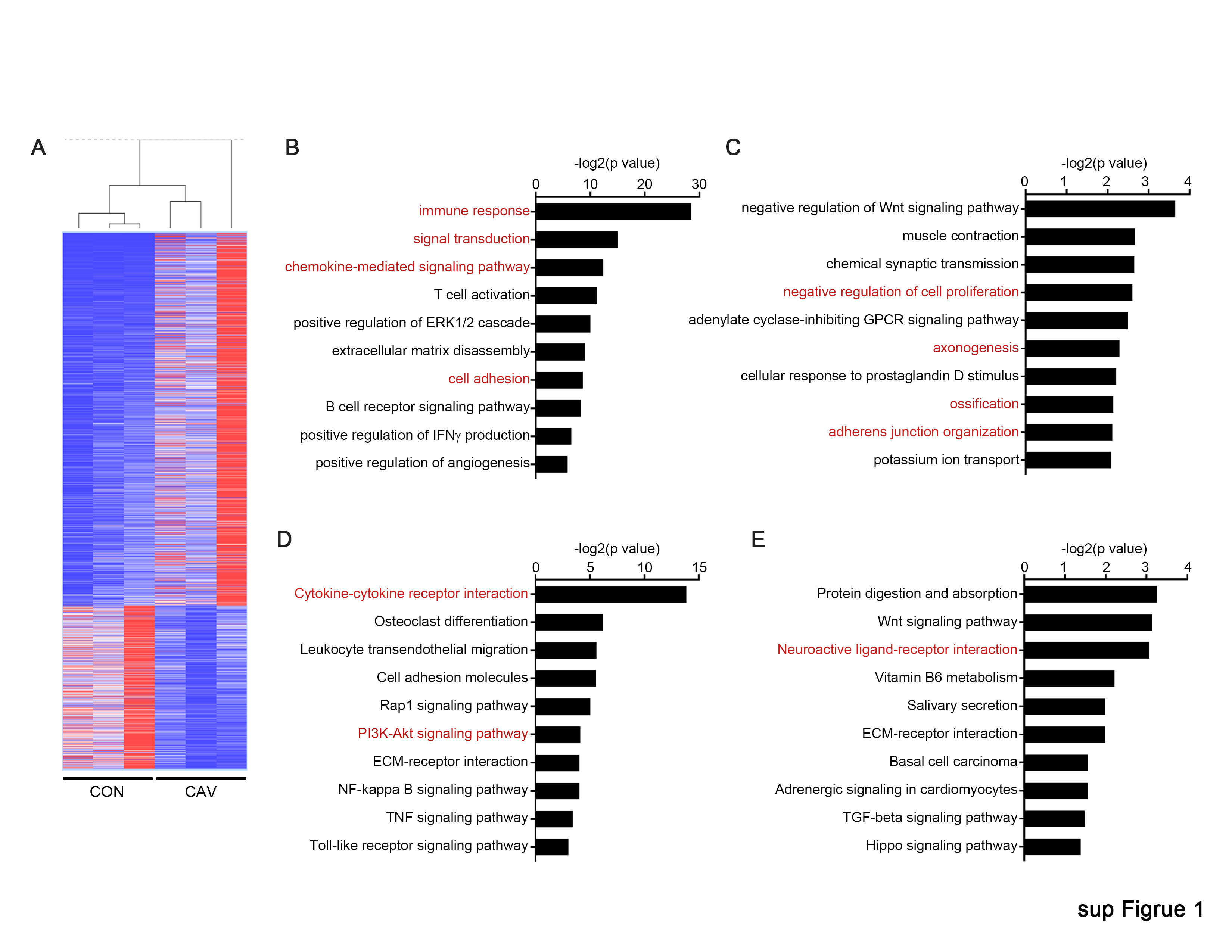

Supplement: Supplementary file 4 [file Image1.TIF]
